# Impact of an ERAS‐Based Surgical Care Bundle Implementation for Preventing Anastomotic Leakage in Minimally Invasive Low Anterior Resection for Rectal Cancer: A Retrospective Cohort Study

**DOI:** 10.1002/wjs.70242

**Published:** 2026-01-23

**Authors:** Koji Tamura, Takaaki Fujimoto, Jinghui Zhang, Kinuko Nagayoshi, Yusuke Mizuuchi, Kohei Horioka, Naoki Ikenaga, Kohei Nakata, Kenoki Ohuchida, Masafumi Nakamura

**Affiliations:** ^1^ Department of Surgery and Oncology, Graduate School of Medical Sciences Kyushu University Fukuoka Japan

**Keywords:** anastomotic leakage, laparoscopic surgery, low anterior resection, rectal cancer, robot‐assisted surgery

## Abstract

**Background:**

Anastomotic leakage (AL) remains a severe complication after low anterior resection (LAR) for rectal cancer, despite advances in minimally invasive (MI) techniques. This study aimed to evaluate the impact of a surgery‐focused care bundle, implemented on an enhanced recovery after surgery (ERAS)‐based perioperative protocol, on preventing AL and improving postoperative outcomes in patients with MI–LAR.

**Methods:**

In this retrospective historically controlled cohort study, a total of 306 patients who underwent MI–LAR between 2011 and 2024 were included. A late‐phase cohort (*n* = 81) receiving the care bundle with an ERAS–based protocol (from September 2019) was compared with a historical early‐phase cohort (*n* = 225). The surgery‐focused care bundle included robot surgery, preoperative oral antibiotics, indocyanine green blood flow evaluation, diverting stoma, transanal drainage tubes, and anastomotic reinforcement. Our institutional ERAS protocol was developed in accordance with the ERAS Society guidelines. Propensity score matching (PSM) was used to adjust for baseline differences between cohorts.

**Results:**

The AL rate significantly decreased from 14.7% (33/225) to 2.5% (2/81) after bundle implementation (*p* < 0.01). Post‐PSM, AL rates remained significantly lower in the late‐phase cohort (18.0% vs. 1.3% and *p* < 0.001). Severe complications (Clavien–Dindo grade ≥ 3) and surgical site infections (SSIs) were also significantly reduced, and no reoperations were required in the late‐phase cohort. Multivariate analysis identified lack of care bundle (odds ratio [OR]: 6.36, 95% confidence interval [CI]: 1.42–28.4, and *p* = 0.01) and male sex (OR: 3.05, 95% CI: 1.24–7.52, and *p* = 0.01) as significant risk factors for AL.

**Conclusions:**

Implementation of a surgery‐focused care bundle, integrated within an ERAS‐based perioperative framework, significantly reduced AL, severe complications, and SSIs after MI‐LAR, suggesting potential long‐term benefits by improving short‐term postoperative outcomes.

## Graphical abstract

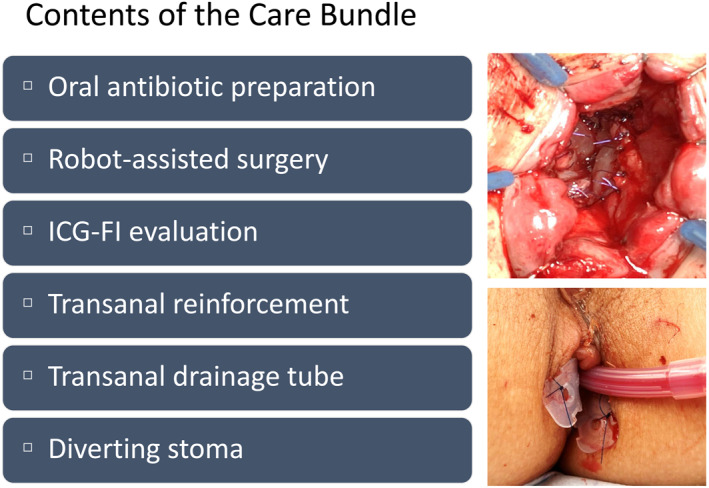

## 1 Introduction

Rectal cancer is a leading cause of cancer‐related death worldwide and curative surgical resection with total mesorectal excision (TME) [1]. Recently, the number of laparoscopic (LAP) surgeries has increased across various surgical specialties [2, 3]. LAP surgery provides an excellent operative field in the narrow pelvic space, facilitating an optimal TME. A randomized controlled trial (RCT) demonstrated that LAP surgery for rectal cancer resulted in rates of local recurrence, disease‐free survival, and overall survival comparable to those of open surgery, with the additional benefit of improved postoperative recovery [4, 5]. Furthermore, recent studies suggest that robot‐assisted (RA) surgery yields superior short‐term and oncological outcomes, compared with LAP surgery for rectal cancer [6, 7, 8].

However, anastomotic leakage (AL) remains a major complication of rectal surgery, particularly in the TME for lower rectal cancer. A low anastomosis during low anterior resection (LAR) inherently carries a higher risk of AL, with reported incidence rates ranging from 7.0% to 28.0% [9, 10, 11, 12]. Despite advancements in minimally invasive surgeries (MISs), AL remains one of the most serious postoperative complications.

A meta‐analysis identified several significant risk factors for AL, including male sex, high body mass index (BMI), large tumor size, preoperative chemotherapy, prolonged operative time, and low anastomotic level [13]. Surgeons must implement the most effective preventive strategies for high‐risk patients. Moreover, postoperative AL not only affects short‐term outcomes and quality of life but may also contribute to disease recurrence in patients with rectal cancer [14]. Consequently, multiple strategies have been introduced to mitigate the risk of AL.

Diverting stomas have long been a widely used approach to reduce AL incidence [9, 12]. Alternatively, some patients who undergo LAR may benefit from transanal drainage tube [15, 16]. Baek et al. reported that transanal reinforcing sutures may reduce the need for a diverting stoma during rectal surgery [17]. In addition, a recent multicenter RCT revealed that the use of indocyanine green fluorescence imaging (ICG‐FI) for rectal cancer significantly reduced AL rates by 4.2% [18]. These measures are typically combined. At our institution, we integrated these strategies into a bundled care approach to prevent AL. To date, few studies have investigated the incidence of AL and its preventive strategy, minimally invasive rectal low‐anterior resection (MI‐LAR). Although several studies have demonstrated the positive effects of bundle care based on the enhanced recovery after surgery (ERAS) protocol, real‐world data focusing on the more technical and surgical aspects—such as the surgery‐focused bundle care described in the present study—remain limited [19, 20].

This study aimed to assess the impact of a surgeryfocused care bundle implementation in preventing AL among patients with rectal cancer undergoing MI‐LAR at a high‐volume center. Additionally, we examined the effects of the care bundle on other short‐term postoperative outcomes, including reoperation rates, surgical site infections (SSIs), and the length of hospital stay.

## 2 Materials and Methods

### 2.1 Study Design

This study was approved by the Ethics Committee of Kyushu University Hospital (approval number: 23336) and was conducted in accordance with the ethical guidelines of the Declaration of Helsinki. This study was reported in accordance with the STROBE statement [21].

The medical records of 621 consecutive patients who were preoperatively diagnosed with rectal cancer or neuroendocrine tumor and underwent rectal anterior resection at the Department of Surgery and Oncology, Kyushu University Hospital between 2011 and 2024 were reviewed. Only patients who underwent LAR in which the anastomotic site was below the peritoneal reflection were included. Some patients underwent intersphincteric resection (ISR), followed by hand‐sewn anastomosis. We included only MIS cases (LAP or RA surgery). When the anastomotic site was located less than 4 cm from the anal verge (AV), the procedure was classified as an ultra‐LAR. The exclusion criteria were as follows: open surgery (non‐MIS), high anterior resection (HAR), abdominoperineal resection, Hartmann's operation, familial adenomatous polyposis, colitis‐associated cancers derived from ulcerative colitis and Crohn's disease, recurrent cancers, other types of malignancies requiring LAR, and simultaneous resection of other synchronous organ diseases.

Tumor location was classified into two categories based on anatomical height: tumors above the peritoneal reflection and those located at or below it were categorized as upper and lower rectal cancer, respectively. Patient outcomes in a late‐phase cohort with care bundle implementation (after September 2019) were compared with those in a historical control group (2011 to August 2019).

The primary outcome was the association between the incidence of postoperative AL and care bundle implementation. AL was defined as a symptomatic postoperative AL (Grades B and C). Secondary outcomes included other perioperative short‐term outcomes, such as reoperation rates, SSIs, and the length of postoperative hospital stay. Postoperative complications were classified according to the Clavien–Dindo (CD) grading system [22]. Demographic and clinical data including age, sex, BMI, American Society of Anesthesiologists‐Physical Status (ASA‐PS) classification, smoking status, comorbidities, preoperative nutritional status, operative findings and procedures, and pathological diagnosis were reviewed. The prognostic nutritional index (PNI) and the geriatric nutritional risk index (GNRI) were calculated as previously described [23, 24].

### 2.2 Perioperative Management Based on Standardized ERAS Protocol

At our institution, a standardized ERAS protocol for colorectal surgery was formally introduced in 2019, in parallel with the implementation of the present surgical care bundle as described previously [25]. The protocol included the following components as shown in Table S1:

1. Preoperative optimization: patient education and counseling, smoking cessation > 4 weeks, optimization of anemia, and nutritional status
2. Antibiotic prophylaxis: intravenous antibiotics administered within 60 min before incision
3. Bowel preparation: a polyethylene glycol‐based mechanical bowel preparation was used together with oral antibiotics (included in the surgical care bundle)
4. Metabolic preparation: standardized fasting and carbohydrate loading
5. Intraoperative management: restrictive fluid therapy and avoidance of excessive fluid administration
6. Analgesia strategy: multimodal analgesia with transition from epidural anesthesia to transverse abdominis plane block
7. Postoperative recovery: early urinary catheter removal, early ambulation on postoperative day (POD) 1, and early oral intake beginning on POD 1.

Basically, all patients in the late‐phase cohort were managed according to the institutional ERAS protocol, although these elements were selectively applied according to the attending surgeon and anesthesiologist before 2019. The surgical care bundle evaluated in this study was designed as a colorectal surgery–specific extension of the institutional ERAS protocol, focusing on technical and surgical preventive measures for AL, such as transanal drainage, reinforcement sutures, and intraoperative ICG‐FI evaluation of perfusion, as described in detail below.

### 2.3 Rectal Surgery–Specific Surgical Care Bundle Implementation

MIS has been the standard surgical approach for the treatment of rectal cancer at our institution since 2006. The presence of abdominal adhesions or prior open abdominal surgery was not considered a contraindication for MIS; however, the final decision between MIS and open surgery was left to the surgeon's discretion as previously described [25].

**FIGURE 1:**
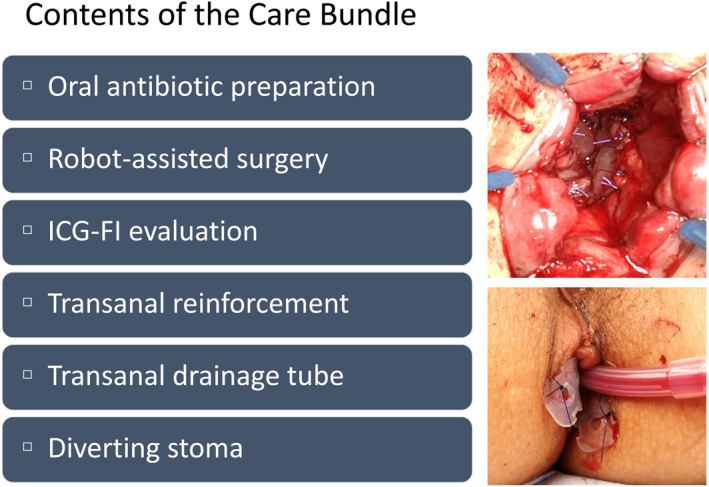
Care bundle contents for preventing anastomotic leakage. Top‐right image shows transanal reinforcement sutures at the anastomotic site. Bottom‐right image shows transanal tube placement. ICG‐FI, indocyanine green fluorescence imaging.

The care bundle components are illustrated in Figure 1. Although the start times for the individual measures varied, the comprehensive care bundle was officially implemented in September 2019. Preoperative oral antibiotic preparation was introduced in mid‐2019, with kanamycin and metronidazole administered orally the day before surgery. As mechanical bowel preparation, magnesium citrate and sennoside were administered to all patients. Regardless of the operative period, a diverting stoma was typically created at the surgeon's discretion for all patients undergoing ultra‐LAR and ISR as well as for high‐risk patients undergoing LAR.

Additionally, a transanal drainage tube was placed in most patients undergoing ultra‐LAR (Figure 1, bottom‐right image). The transanal drainage tube was typically removed on POD 3 when stool or fluid output was minimal. In most cases, removal occurred around POD 5, followed by oral intake initiation. Patients with frequent loose stools occasionally required longer placement. Early ambulation was encouraged even with the tube in place, although prolonged sitting position was avoided.

Since 2019, ICG‐FI has been used selectively for colonic perfusion assessment in rectal surgery. Following care bundle implementation, ICG‐FI evaluation during anastomosis became routine, regardless of the surgeon. In late 2020, transanal reinforcement sutures were introduced at the anastomotic site in patients with a lower anastomosis. Typically, circumferential anastomotic reinforcement is performed using 4–0 absorbable polydioxanone sutures, with approximately 16–20 stitches (Figure 1, top‐right image).

### 2.4 Statistical Analysis

Historical control and propensity score matching (PSM) were used to minimize bias and estimate the effect of care bundle implementation. Comparisons between cohorts were performed using the chi‐squared test or Mann–Whitney *U* test as appropriate. PSM analysis was conducted to minimize the influence of potential confounders and selection bias before and after care bundle implementation. Propensity scores were generated based on patient characteristics (age, sex, BMI, diabetes mellitus [DM], and smoking status), tumor location, presence of lateral lymph node dissection (LLND), use of neoadjuvant treatment, and pathological stage using binary logistic regression. One‐to‐one matching between the two cohorts was achieved with a caliper width of 0.2 standard deviations of the logit of the estimated propensity score. Univariate analyses were performed to identify the risk factors for AL. All variables with a *p* value < 0.2 in the univariate analysis were included in the multivariate logistic regression analysis. There were no missing data for the variables used in the main analyses. Statistical significance was set at *p* < 0.05. All statistical analyses were performed using JMP Pro version 17.0.0 (SAS Institute Inc., Tokyo, Japan).

## 3 Results

### 3.1 Patient Characteristics

**FIGURE 2:**
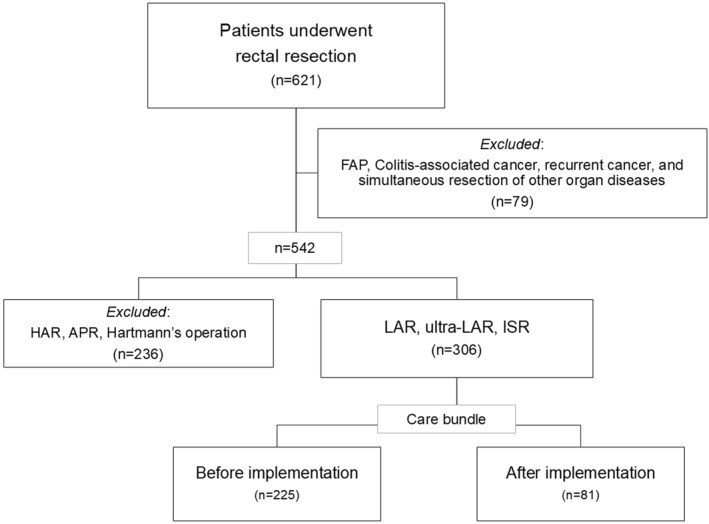
Patient flowchart. The medical records of 621 consecutive patients who were preoperatively diagnosed with rectal cancer or rectal neuroendocrine tumor/carcinoma and underwent surgical resection were reviewed. A total of 306 patients met the inclusion criteria and were categorized into cohorts based on the implementation of care bundle. APR, abdominoperineal resection; FAP, familial adenomatous polyposis; HAR, high‐anterior resection; ISR, intersphincteric resection; LAR, low‐anterior resection.

Figure 2 shows the flowchart of patient inclusion. In total, 306 patients who underwent MI‐LAR met the inclusion criteria. Among them, 81 (26.5%) were included in the late‐phase cohort (care bundle group), whereas 225 (73.5%) were included in the early‐phase cohort (historical control without care bundle implementation) (Figure 2). There were no significant differences between the cohorts in terms of age, sex, ASA‐PS, BMI, smoking status, PNI, GNRI, use of neoadjuvant treatment, tumor location, tumor distance from the AV, surgical method, or tumor stage (Table 1). The prevalence of DM was significantly higher in the late‐phase cohort than in the early‐phase cohort (4.9% vs. 15.1% and *p* = 0.02).

**TABLE 1:** Patients' characteristics before and after care bundle implementation.

Total (*n* = 306)		Care bundle		*p* value
		Before (*n* = 225)	After (*n* = 81)	
Age		63.4 ± 12.2	63.8 ± 11.0	0.95
Sex	Male	126 (56.0%)	47 (58.0%)	0.75
	Female	99 (44.0%)	34 (42.0%)	
ASA—PS	1	49 (21.8%)	17 (21.0%)	0.99
	2	160 (71.1%)	58 (71.6%)	
	3	16 (7.1%)	6 (7.4%)	
BMI	(kg/m^2^)	22.0 (14.8–35.1)	22.4 (15.4–38)	0.27
Smoking		45 (20.0%)	16 (19.8%)	0.25
DM		34 (15.1%)	4 (4.9%)	**0.02**
PNI		50.4 (28.9–68.5)	48.9 (34.8–58.9)	0.13
GNRI		104.3 (70.9–132.3)	103.8 (79.7–134.5)	0.84
Neoadjuvant treatment	Yes/no	38 (16.9%)/187 (83.1%)	15 (18.5%)/66 (81.5%)	0.73
Tumor location	Upper	101 (44.9%)	34 (42.0%)	0.65
	Lower	124 (55.1%)	47 (58.0%)	
AV	(cm)	7.0 (2.0–15.0)	7.0 (2.0–17.0)	0.60
Operation	LAR	118 (52.4%)	40 (49.3%)	0.64
	Ultra‐LAR/ISR	74 (32.9%)/33 (14.7%)	39 (48.2%)/2 (2.5%)	
LLND	Yes/no	38 (16.9%)/187 (83.1%)	6 (7.4%)/75 (92.6%)	**0.04**
Operation time	(min)	353 (172–884)	329 (187–812)	0.07
Blood loss	(g)	50 (1–2138)	21 (1–630)	**<** **0.0001**
pStage	0/1	8 (3.6%)/84 (37.3%)	0 (0%)/29 (35.8%)	0.26
	2	46 (20.4%)	11 (13.6%)	
	3	57 (25.3%)	30 (37.0%)	
	4	20 (8.9%)	7 (8.6%)	
	pCR	3 (1.3%)	1 (1.2%)	
	NET/NEC	6 (2.7%)/1 (0.4%)	3 (3.7%)/0 (0%)	

Abbreviations: ASA‐PS, American society of anesthesiologists‐physical status; AV, anal verge; BMI, body mass index; DM, diabetes mellitus; GNRI, geriatric nutritional risk index; ISR, intersphincteric resection; LAR, low anterior resection; LLND, lateral lymph node dissection; NEC, neuroendocrine carcinoma; NET, neuroendocrine tumor; pCR, pathological complete response after neoadjuvant chemotherapy; PNI, prognostic nutritional index.

Patients in the late‐phase cohort underwent LLND significantly less frequently compared with those in the early‐phase cohort (7.4% vs. 16.9%, *p* = 0.04). Additionally, intraoperative blood loss was significantly lower in the late‐phase cohort than in the early‐phase cohort (21 [1–630] vs. 50 [1–2138] grams [g]; *p* < 0.0001, Table 1). Although the operative time was shorter in the late‐phase cohort than in the early‐phase cohort, the difference was not significant (329 [187–812] vs. 353 [172–884] min and *p* = 0.07). The PSM results are presented in Table S2. After adjusting for patient and operative factors, the difference in intraoperative blood loss between the two cohorts remained significant (20.5 [1–630] vs. 43.5 [1–806] g and *p* < 0.01).

### 3.2 Compliance With the Care Bundle

The compliance rates for each component of the care bundle are listed in Table 2. In the late‐phase cohort, 88.9% (72/81) of the patients underwent RA surgery compared with only 6.7% (15/225) of the patients in the early‐phase cohort (*p* < 0.0001). Oral antibiotic preparation was performed in 86.4% (70/81) of the late‐phase cohort patients versus only 0.4% (1/225) of the early‐phase cohort patients (*p* < 0.0001). Almost all patients (98.8%) in the late‐phase cohort underwent intraoperative ICG‐FI evaluation, whereas only 11.1% (10/225) of the patients in the early‐phase cohort (*p* < 0.0001) underwent this evaluation. The rate of diverting stoma creation did not differ significantly between the cohorts. Transanal drainage tube placement was significantly more common in the late‐phase cohort than in the early‐phase cohort (81.5% [66/81] vs. 50.7% [114/225]; *p* < 0.0001). Transanal reinforcement suturing was performed significantly more frequently in the late‐phase cohort than in the early‐phase cohort (38.0% vs. 0.5% and *p* < 0.0001). Among the 39 patients in the late‐phase cohort who underwent ultra‐LAR, 29 received reinforcement sutures. Notably, approximately half (14 patients) of them did not require creation of diverting stoma and none experienced AL (data not shown). Moreover, all patients in the late‐phase cohort who underwent ultra‐LAR without transanal reinforcement underwent diverting ileostomies.

**TABLE 2:** Compliance rate of care bundle.

Total (*n* = 306)		Care bundle		*p* value
		Before (*n* = 225)	After (*n* = 81)	
Robot‐assisted surgery		15/225 (6.7%)	72/81 (88.9%)	**<** **0.0001**
OABP		1/225 (0.4%)	70/81 (86.4%)	**<** **0.0001**
ICG evaluation		10/225 (4.4%)	80/81 (98.8%)	**<** **0.0001**
Diverting stoma	Total	83/225 (36.9%)	33/81 (40.7%)	0.54
	LAR	11/118 (9.3%)	7/40 (17.5%)	0.16
	Ultra‐LAR	40/74 (54.1%)	25/39 (64.1%)	0.30
	ISR	32/33 (97.0%)	1/2 (50.0%)	**0.03**
Transanal tube	Total	114/225 (50.7%)	66/81 (81.5%)	**<** **0.0001**
	LAR	62/118 (52.5%)	35/40 (87.5%)	**0.0001**
	Ultra‐LAR	51/74 (68.9%)	31/39 (79.5%)	0.23
	ISR	1/33 (3.0%)	0/2 (0%)	**<** **0.01**
Transanal reinforcement	LAR/ultra‐LAR	1/192 (0.5%)	30/79 (38.0%)	**<** **0.0001**
	Ultra‐LAR	1/74 (1.4%)	29/39 (74.4%)	**<** **0.0001**

Abbreviations: ICG, indocyanine green; ISR, intersphincteric resection; LAR, low anterior resection; OABP, oral antibiotic preparation.

### 3.3 Comparison of Postoperative Short‐Term Outcomes

The short‐term outcomes of the late‐phase cohort patients were compared with those of the early‐phase cohort patients (Table 3). Overall, AL occurred in 35 patients (11.4%); the list of these patients is provided in Table S3. The AL rate was significantly lower in the late‐phase cohort than in the early‐phase cohort (2.5% vs. 14.7% and *p* < 0.01). This difference remained significant after PSM (1.3% vs. 18.0% and *p* < 0.001). The rate of CD grade ≥ 3 complications was significantly lower in the late‐phase cohort than in the early‐phase cohort, both before (16.0% vs. 6.2% and *p* = 0.03) and after (15.4% vs. 5.1% and *p* = 0.03) PSM. There was no significant difference in the reoperation rate between the two cohorts; however, no reoperation was required after the care bundle implementation. The SSI rate was significantly lower in the late‐phase cohort than in the early‐phase cohort, both before (8.6% vs. 19.6% and *p* = 0.02) and after (7.7% vs. 21.8% and *p* = 0.01) PSM.

**TABLE 3:** Comparison of postoperative short‐term outcomes before and after propensity score matching.

Total (*n* = 306)	Care bundle		*p* value
	Before (*n* = 225)	After (*n* = 81)	
CD ≥ 2	75 (33.3%)	27 (33.3%)	1.00
CD ≥ 3	36 (16.0%)	5 (6.2%)	**0.03**
Anastomotic leakage	33 (14.7%)	2 (2.5%)	**<** **0.01**
SSI^a^	44 (19.6%)	7 (8.6%)	**0.02**
Reoperation	9 (4.0%)	**0 (0%)**	0.07
Hospital stay (days)	14 (7–152)	16 (9–45)	0.29

After PSM (*n* = 156)	Before (*n* = 78)	After (*n* = 78)	*p* value
CD ≥ 2	29 (37.2%)	25 (21.1%)	0.50
CD ≥ 3	12 (15.4%)	4 (5.1%)	**0.03**
Anastomotic leakage	14 (18.0%)	1 (1.3%)	**<** **0.001**
SSI^a^	17 (21.8%)	6 (7.7%)	**0.01**
Reoperation	3 (3.9%)	**0 (0%)**	0.08
Hospital stay (days)	14 (8–110)	16 (9–45)	0.17

Abbreviations: CD, Clavien–Dindo grading; PSM, propensity score matching; SSI, surgical site infection.

^a^ Including anastomotic leakage.

There were no significant differences between the cohorts in terms of CD grade ≥ 2 complications or the length of postoperative hospital stay. These findings remained consistent after PSM.

### 3.4 Risk Factors for AL

The clinical characteristics of the 35 patients with AL were compared with those of 271 patients without AL using univariate and multivariate analyses (Table 4). There were no significant differences in age, ASA‐PS score, BMI, smoking status, PNI, GNRI, tumor location, tumor distance from the AV, presence of LLND, or pathological stage. Lack of care bundle implementation (*p* < 0.01), male sex (*p* < 0.01), DM (*p* = 0.01), use of neoadjuvant treatment (*p* = 0.02), operation time (*p* < 0.01), and intraoperative blood loss (*p* < 0.001) were correlated with the AL rate. Patients with AL experienced a significantly prolonged postoperative hospital stay than did those without AL (43 [16–152] vs. 14 [7–110] days and *p* < 0.0001).

**TABLE 4:** The relationships between anastomotic leakage and clinical factors.

Total (*n* = 306)		AL+	AL−	Univariate	Multivariate	
		(*n* = 35)	(*n* = 271)	*p* value	OR (95% CI)	*p* value
Care bundle	Before	33 (94.3%)	192 (70.8%)	**<** **0.01**	**6.87 (1.97–43.6)**	**0.01**
	After	2 (5.7%)	79 (29.2%)		ref	
Age	(years)	60.1 ± 10.2	64.0 ± 12.0	0.05		
Sex	Male	28 (80.0%)	145 (53.5%)	**<** **0.01**	**3.26 (1.41–8.50)**	**<** **0.01**
	Female	7 (20.0%)	126 (46.5%)		ref	
ASA—PS	1	8 (22.9%)	58 (21.4%)	0.93		
	2	25 (71.4%)	193 (71.2%)			
	3	2 (5.7%)	20 (7.4%)			
BMI	(kg/m^2^)	23.0 (17.3–30.4)	21.8 (14.8–38.0)	0.14		
Smoking	Yes	8 (22.9%)	53 (19.6%)	0.65		
	No	27 (77.1%)	218 (80.4%)			
DM	Yes	9 (25.7%)	29 (10.7%)	**0.01**	2.16 (0.85–5.17)	0.10
	No	26 (74.3%)	242 (89.3%)		ref	
PNI		50.0 (35.4–60.6)	50.2 (28.9–68.5)	0.52		
GNRI		101.9 (73.6–126.7)	104.3 (70.9–134.5)	0.87		
Neoadjuvant treatment	Yes	11 (31.4%)	42 (15.5%)	**0.02**	**2.55 (1.08–5.80)**	**0.03**
	No	24 (68.6%)	229 (84.5%)			
Tumor location	Upper	13 (37.1%)	122 (45.0%)	0.38		
	Lower	22 (62.9%)	149 (55.0%)			
AV	(cm)	6.0 (2.0–14.0)	7.0 (2.0–17.0)	0.2		
Operation	LAR	16 (45.7%)	142 (52.4%)	0.72		
	Ultra‐LAR/ISR	14 (40.0%)/5 (14.3%)	99 (36.5%)/30 (11.1%)			
LLND	Yes	9 (24.7%)	35 (12.9%)	0.06		
	No	26 (74.3%)	236 (87.1%)			
Operation time	(min)	438 (172–884)	338 (178–831)	**<** **0.01**		
Blood loss	(mL)	80 (1–900)	44 (1–2138)	**<** **0.001**		
pStage	0/1	0 (0%)/12 (34.3%)	6 (2.2%)/101 (37.3%)	0.76		
	2	6 (17.1%)	51 (18.9%)			
	3	8 (22.9%)	79 (29.2%)			
	4	7 (20.0%)	22 (8.1%)			
	pCR	1 (2.9%)	3 (1.1%)			
	NET/NEC	1 (2.9%)/0 (0%)	8 (3.0%)/1 (0.4%)			
Hospital stay	(days)	43 (16–152)	14 (7–110)	**<** **0.0001**		

Abbreviations: AL, anastomotic leakage; ASA‐PS, American Society of Anesthesiologists‐Physical Status; AV, anal verge; BMI, body mass index; CI, confidence interval; DM, diabetes mellitus; GNRI, geriatric nutritional risk index; ISR, intersphincteric resection; LAR, low anterior resection; LLND, lateral lymph node dissection; NEC, neuroendocrine carcinoma; NET, neuroendocrine tumor; OR, odds ratio; pCR, pathological complete response after neoadjuvant chemotherapy; PNI, prognostic nutritional index.

Multivariate analysis identified lack of care bundle implementation (odds ratio [OR]: 6.36, 95% confidence interval [CI]: 1.42–28.4, and *p* = 0.01) and male sex (OR: 3.05, 95% CI: 1.24–7.52, and *p* = 0.01) as independent risk factors for AL.

## 4 Discussion

This observational historical control study at a high‐volume center is, to the best of our knowledge, among the few to evaluate the advantages of a technically focused care bundle based on ERAS principles in improving the short‐term outcomes, including reducing postoperative AL incidence and the rates of reoperation, CD grade ≥ 3 complications, and SSIs in patients with lower rectal cancer undergoing MI‐LAR. A multicenter RCT previously reported that AL is associated with increased local recurrence and decreased disease‐free survival [26]. Therefore, our care bundle strategy may contribute not only to short‐term outcomes but also to the long‐term oncological outcomes of patients after rectal cancer surgery. Initially, the adoption of LAP surgery increased with decreases in intraoperative and postoperative complications [3]. However, particularly in rectal surgery, RA surgery has now surpassed LAP surgery in global adoption at high‐volume centers, owing to its superior maneuverability and visualization. A recent multicenter RCT showed that RA surgery improved oncological resection quality, reduced surgical trauma, and enhanced postoperative recovery in patients with lower rectal cancer [6]. Additionally, Obatake et al. reported that, compared with LAP surgery, RA surgery is a safer and more feasible approach for rectal cancer, offering greater sphincter preservation, particularly in low rectal cancer [27]. In our study, the number of ISR cases were lower in the late‐phase cohort, suggesting that RA surgery contributes significantly to sphincter preservation.

However, a previous meta‐analysis did not show significant advantages of RA surgery over LAP surgery in reducing major complications, including AL, during rectal cancer treatment [28]. In our study, compared with the early‐phase cohort, RA surgery was significantly more prevalent in the late‐phase cohort, which coincided with a lower AL rate. Unlike previous meta‐analyses that included HAR cases, our study focused solely on patients with LAR, which may explain the observed differences in the AL rates. In a multicenter RCT (REAL trial), although not significant, the AL rate for RA surgery (5.1%) for middle to low rectal cancer was lower than that for LAP surgery (8.2%; *p* = 0.057 and 95% CI: −6.5–0.1) [6]. Based on these findings, RA surgery may have contributed to reduced AL rates and could be recommended as part of a bundled strategy for rectal cancer treatment.

A major difference between the early‐ and late‐phase cohorts was the significantly higher use of transanal reinforcement sutures, particularly in patients with ultra‐LAR. This technique has been reported as an effective AL prevention strategy and may reduce the need for diverting ileostomy [17]. Enomoto et al. demonstrated that stapled anastomosis with transanal reinforcement resulted in a lower AL rate compared with coloanal handsewn anastomosis after transanal TME [29]. In our study, approximately half of the patients who underwent ultra‐LAR with transanal reinforcement sutures did not require creation of a diverting stoma and none developed AL. Even in cases in which an incomplete stapled ring was observed during stapled anastomosis, reinforcement sutures ensured anastomotic security. Given that lower anastomoses are easier to access transanally, this technique should be considered the standard approach for AL prevention.

Transanal drainage tube placement is another key component of the care bundle. We have previously conducted a meta‐analysis to demonstrate the effectiveness of transanal drainage tube after LAR [15]. Although universal transanal drainage tube placement remains controversial, transanal drainage tube significantly lowers the AL and reoperation rates in patients without diverting stoma. Considering the low risk of adverse events associated with transanal drainage tube, it is a promising alternative for diverting stoma creation. Further studies are required to identify the optimal candidates for transanal drainage tube placement.

Insufficient blood perfusion is a major concern during colorectal anastomosis. A phase‐3 RCT demonstrated that ICG‐FI significantly reduced the AL rates by 4.2% in MIS for rectal cancer [18]. Given its proven efficacy, the ICG‐FI was integrated into our care bundle. However, AL is likely influenced by multiple factors beyond perfusion alone, including high intraluminal pressure, excessive anal sphincter spasms, anastomotic tension, and other patient‐related factors [30]. The RCT‐reported AL rate (7.6%) in the ICG group was slightly higher than that reported in our study. This finding suggests that our care bundle may address additional risk factors for AL beyond perfusion.

A recent multicenter RCT demonstrated that, in patients undergoing elective rectal resection, mechanical bowel preparation combined with oral antibiotic preparation significantly reduced overall postoperative complications, including AL, compared with mechanical bowel preparation alone (5.8% vs. 13.5%; OR: 0.39). However, in this study, > 75% of the patients underwent open surgery [31]. A meta‐analysis also highlighted the potential benefits of oral antibiotic preparation; however, most of the included studies focused on open surgery and colonic resections [32]. In our study, the AL and SSI rates significantly decreased following care bundle implementation, suggesting that oral antibiotic preparation may also contribute to AL reduction in MI‐LAR. Further RCTs are required to confirm their role in MI‐rectal surgery.

RCTs and meta‐analyses have demonstrated that a defunctioning stoma reduces clinically relevant AL and is recommended for low rectal cancer surgery [12, 33]. However, these studies primarily involved open surgery and excluded patients who underwent MIS. With MIS becoming the preferred approach for low rectal cancer at high‐volume centers, the role of diverting stomas in MI‐LAR remains unclear. Our institution has long used diverting stomas in patients with ultra‐LAR, ISR, and high‐risk LAR, and this strategy has remained unchanged between cohorts. A large‐scale multicenter RCT focusing on MIS is warranted to clarify its necessity in modern surgical practice.

These results are consistent with recent ERAS Society recommendations emphasizing oral antibiotics combined with mechanical bowel preparation, restrictive fluid therapy, and early mobilization as core elements of colorectal ERAS pathways [20]. In our institution, the ERAS protocol was formally adopted in 2019, and the surgical care bundle was implemented concurrently as a rectal surgery–specific enhancement focusing on technical measures to improve anastomotic safety. Although the ERAS protocol itself may have contributed to the overall improvement in postoperative outcomes, our findings suggest that the addition of this surgery‐focused care bundle further enhanced anastomotic security and reduced postoperative complications. This highlights the potential synergistic effect of combining ERAS‐based perioperative management with procedure‐specific surgical interventions.

The present study has some limitations. First, it was a historically controlled single‐center study, which may have introduced institutional and selection bias despite the use PSM. Differences in surgeons between the two groups may have influenced the outcomes, and the involvement of surgeon‐related bias cannot be ruled out. To enhance generalizability, a prospective multicenter cohort study based on standardized protocols is required. Second, although the timing of surgical care bundle implementation was predetermined, the start time of individual AL prevention measures varied, potentially introducing bias. Moreover, the institutional ERAS program was also gradually implemented beginning in early 2019, with some ERAS‐related elements already being practiced even before formal adoption. Because of the retrospective nature of this study and the variable timing of ERAS component introduction, it was not feasible to reliably extract or quantify adherence rates for each individual ERAS item. A prospective RCT with a standardized protocol could minimize this variability. Third, long‐term outcomes were not assessed. After excluding patients with a follow‐up period of less than 5 years or those with stage IV disease, the remaining number of patients in the late‐phase cohort was small. When further stratified according to stage, the number of patients in each cohort was significantly reduced, leading to a high likelihood of bias. Further studies are necessary to evaluate the long‐term impacts of care bundle implementation. It should be noted that the annual number of patients undergoing LAR was slightly lower in the later period than in the earlier period. Although the impact of the COVID‐19 pandemic cannot be excluded, it may also reflect changes in regional surgical practice. Several colorectal surgeons trained at our institution subsequently moved to affiliated mid‐volume hospitals, where LAR with low anastomosis has been safely performed. This redistribution of surgical cases may have contributed to the observed decrease in rectal surgery volume at our center.

Since the implementation of bundled care, the AL rate has remained as low as 2.5% (two cases among 81 patients in the late‐phase cohort) in patients undergoing LAR, which is an exceptionally favorable outcome, compared with global data. Although individual AL prevention measures may not significantly reduce the AL rate, their combined implementation within a structured care bundle is likely to yield substantial improvements in postoperative outcomes.

In conclusion, implementing a surgery‐focused care bundle integrated within an ERAS‐based perioperative framework for patients undergoing LAR may reduce the incidence of AL and improve the short‐term postoperative outcomes. Transanal drainage tube placement and transanal reinforcement sutures could potentially serve as alternatives to diverting stoma creation.

## Author Contributions

**Koji Tamura:** conceptualization, writing – original draft, writing – review and editing, methodology, software, formal analysis, investigation, data curation, project administration. **Takaaki Fujimoto:** data curation, formal analysis, resources, writing – original draft. **Jinghui Zhang:** data curation, resources. **Kinuko Nagayoshi:** investigation, data curation. **Yusuke Mizuuchi:** data curation, resources, validation. **Kohei Horioka:** data curation, resources. **Naoki Ikenaga:** investigation, data curation. **Kohei Nakata:** data curation, investigation, supervision. **Kenoki Ohuchida:** conceptualization, supervision, writing – review and editing. **Masafumi Nakamura:** supervision, writing – review and editing.

## Funding

The authors have nothing to report.

## Ethics Statement

The protocol for this research project has been approved by a suitably constituted Ethics Committee of Kyushu University and it conforms to the provisions of the Declaration of Helsinki. Approval number: 23336.

## Consent

Informed consent was obtained through an opt‐out form on the website.

## Conflicts of Interest

Drs. Koji Tamura, Takaaki Fujimoto, Jinghui Zhang, Kinuko Nagayoshi, Yusuke Mizuuchi, Kohei Horioka, Naoki Ikenaga, Kohei Nakata, Kenoki Ohuchida, and Masafumi Nakamura have no conflicts of interest or financial ties to disclose.

## Supporting information


**Table S1:** Perioperative management based on standardized ERAS protocol.

**Table S2:** Patients' characteristics before and after care bundle implementation after propensity score matching.

**Table S3:** Patients with postoperative anastomotic leakage.

## Data Availability

The data that support the findings of this study are available from the corresponding author upon reasonable request.
